# Using systems thinking to identify workforce enablers for a whole systems approach to urgent and emergency care delivery: a multiple case study

**DOI:** 10.1186/s12913-016-1616-y

**Published:** 2016-08-09

**Authors:** Kim Manley, Anne Martin, Carolyn Jackson, Toni Wright

**Affiliations:** England Centre for Practice Development, Faculty of Health and Wellbeing, Canterbury Christ Church University, North Holmes Road CT1 1QU, Canterbury, UK

**Keywords:** Urgent and emergency care, Whole systems working, Leadership, Workforce development, Multiple case study, Facilitation, Work based learning, Integrated competence framework

## Abstract

**Background:**

Overcrowding in emergency departments is a global issue, which places pressure on the shrinking workforce and threatens the future of high quality, safe and effective care. Healthcare reforms aimed at tackling this crisis have focused primarily on structural changes, which alone do not deliver anticipated improvements in quality and performance. The purpose of this study was to identify workforce enablers for achieving whole systems urgent and emergency care delivery.

**Methods:**

A multiple case study design framed around systems thinking was conducted in South East England across one Trust consisting of five hospitals, one community healthcare trust and one ambulance trust. Data sources included 14 clinical settings where upstream or downstream pinch points are likely to occur including discharge planning and rapid response teams; ten regional stakeholder events (*n* = 102); a qualitative survey (*n* = 48); and a review of literature and analysis of policy documents including care pathways and protocols.

**Results:**

The key workforce enablers for whole systems urgent and emergency care delivery identified were: clinical systems leadership, a single integrated career and competence framework and skilled facilitation of work based learning.

**Conclusions:**

In this study, participants agreed that whole systems urgent and emergency care allows for the design and implementation of care delivery models that meet complexity of population healthcare needs, reduce duplication and waste and improve healthcare outcomes and patients’ experiences. For this to be achieved emphasis needs to be placed on holistic changes in structures, processes and patterns of the urgent and emergency care system. Often overlooked, patterns that drive the thinking and behavior in the workplace directly impact on staff recruitment and retention and the overall effectiveness of the organization. These also need to be attended to for transformational change to be achieved and sustained. Research to refine and validate a single integrated career and competence framework and to develop standards for an integrated approach to workplace facilitation to grow the capacity of facilitators that can use the workplace as a resource for learning is needed.

**Electronic supplementary material:**

The online version of this article (doi:10.1186/s12913-016-1616-y) contains supplementary material, which is available to authorized users.

## Background

Overcrowding in emergency departments is a global issue, which places pressure on the shrinking workforce and threatens the future of high quality, safe and effective care [1–4]. Despite much analysis, there is no single factor to explain this trend or variations in healthcare outcomes [5, 6]. There is general consensus that whole systems working is needed to tackle overcrowding in emergency departments [7, 8], but healthcare reforms tend to focus primarily on structural changes which alone, do not deliver anticipated improvements in quality and performance [9]. Emphasis on processes and structures overlooks patterns manifested in relationships, beliefs, traditions, power, values and assumptions, which form workplace culture and are highly influential in adopting change in healthcare systems [10]. Research indicates that investments in healthcare fail to yield full benefit due to difficulties of creating and maintaining an effective, efficient and motivated workforce [11]. This study aimed to identify workforce enablers for achieving whole systems urgent and emergency care across one Trust consisting of five hospitals, one community healthcare trust and one ambulance trust in South East England.

Urgent and emergency care refers to the range of healthcare services available to people who need medical advice, diagnosis and/or treatment quickly and unexpectedly [12]. The rising demand for urgent and emergency care in many countries is attributed to a complex mix of changing demographic, health, economic, social and system design factors [3, 7]. Alternative primary care services appear not only to increase overall demand, but may also create a fragmented system which generates disorder among the public, general practitioners and other referral services about how and where to access care [13, 14]. These have bearing on the effectiveness of urgent and emergency care systems, which rely on collaborative partnerships with other services and specialties to implement integrated care pathways and improve patient outcomes [15].

Pressures arising from increased workloads and limited resources are diminishing the desirability of careers in emergency medicine. This is exemplified by fewer practitioners choosing to pursue a career in emergency medicine citing poor working conditions, work-life balance, a target-driven culture, and the lack of 24-hour consultant support as reasons for their lack of desire to engage in this work [16]. As a result, healthcare providers are unable to recruit to substantive posts and current stopgap solutions are unsustainable in the longer term [17]. In the United Kingdom (UK) for example, there is a high dependency on the use of temporary ‘locum’ staff without specialist knowledge of emergency medicine, who need more support from doctors in training than they are able to return [18]. The UK Centre for Workforce Intelligence [19] predicted an expenditure of approximately six billion pounds (nine billion dollars) on emergency care consultant fees by 2020. The Global Health Alliance [20] suggests integrated and coordinated approaches to address capacity management as well as issues that affect production, deployment, absorption, performance, and motivation of the healthcare workforce.

Whole system approaches to urgent and emergency care have garnered attention as strategies to alleviate overcrowding in emergency departments (ED) [7, 8] but there is limited evidence about enabling the workforce to implement whole systems working. Existing whole systems workforce development models [21, 22] fail to draw on the workplace as the main resource for learning and do not demonstrate the contribution of interdependent partners to the whole. Attwood et al. [23] focus on overcoming structural and processes barriers to work effectively as part of a wider system. Prat et al. posit that complex issues are better tackled as whole and interconnected rather than actionable parts. This involves a combination of theory and practical methods of working across boundaries [24]. This paper reports on key system and workforce enablers for developing a workforce capable of delivering consistently high quality, person centered, safe and effective care and promoting smooth transition of the patient’s journey across care settings.

## Methods

### Design

A descriptive multiple case study design was used to clarify gaps and pinch points in the various contexts of the urgent and emergency care pathway across primary/community, secondary and tertiary settings. This design facilitates empirical investigation of phenomenon within real life contexts to answer ‘how’ and ‘why’ questions, especially when the boundaries between the phenomenon and the contexts are not evident [25]. A multiple case study was deemed suitable to explore differences and similarities in the various contexts of urgent and emergency care delivery guided by two research questions:

- How do we solve the current workforce crisis in emergency departments creatively to promote sustainable transformational change?
- What should the urgent and emergency workforce of the future look like?

Data collection and analysis were informed by systems thinking which provides a rational process for mapping and understanding relationships to address complex issues in a holistic way. Checkland defines a system as a complex entity at the core of which is the concept of a whole that can adapt and survive within limits in a changing environment [26]. The whole entity in this study is the urgent and emergency care system embracing partners from primary/community, secondary and tertiary care that contribute to wider processes of the urgent and emergency care pathway. A whole system’s strength is contingent on the functioning of contributing partners and ongoing dynamic feedback [27].

This study was based on the concept that achieving transformational change in complex systems also requires attention to patterns that drive thinking and behavior in order to promote a sense of shared responsibility that mirrors ways of working among interdependent partners [10]. Systems thinking provided the framework for exploring perceived or experienced gaps and pinch points in the urgent and emergency care system and guided the generation of synergistic options relevant to the whole system that is greater than the sum of individual parts.

### Data collection

Data were collected from multiple sources using the fourth generation stakeholder evaluation approach [28]. The method involved identifying urgent and emergency care stakeholders, exploring stakeholders’ claims, concerns and issues about the urgent and emergency care pathway; and seeking consensus among stakeholders about emerging themes through discussion. Multiple sources of evidence are highly complementary to cross case synthesis to triangulate information from each data source [25, 29]. Data collection was also guided by collaborative, inclusive, participatory principles of practice development aimed to develop a shared purpose underlined by person-centered values and effective workplace cultures that enable individuals and teams to flourish [30].

Four methods were used to gather qualitative data to gain clear understanding of issues emerging in urgent and emergency care. Firstly, a review of evidence relating to urgent and emergency care delivery including care pathways and protocols was undertaken to understand international and national workforce implications. The review was guided by six questions:

- What is urgent and emergency care?
- What are the redesign, innovation and policy issues?
- What are the required standards?
- What are the required competences?
- What factors enable or inhibit these?
- What are service user perspectives on urgent care?

Competence was defined as acquiring and using evidence-based scientific and humanistic knowledge and skill in the application of therapeutic interventions in the practice setting [31].

Secondly data were collected from ten regional stakeholder events held in South East England across one Trust consisting of five hospitals, one community healthcare trust and one ambulance trust. The events were widely publicized using both electronic and paper flyers which included the research questions. The stakeholder events were facilitated using a claims, concerns, and issues approach [28] to pave way for insights on:

- Understanding of urgent and emergency care
- Ultimate purpose of urgent and emergency care
- How purpose is achieved
- Current urgent and emergency care pathways
- Potential or future urgent care pathways
- Enablers and inhibitors
- Key competences required for current and future integrated urgent and emergency care
- Perceived gaps in pathway competence
- Key roles in urgent and emergency care
- What would happen in an effective integrated urgent and emergency care system and how effectiveness would be recognized? This specific question drew on the concept of ‘the miracle question’ from solution focused approaches to envisage options for achieving an effective system in the future [32].
- Other considerations

At each stakeholder event, discussion points and other verbal contributions were noted on separate flipcharts for each question to enable collaborative data analysis. Some participants gave accounts of their experience with or within the service to demonstrate the gaps and pinch points in the service and or in the workforce. These accounts were themed but also maintained as case examples (Additional file 1). Stakeholder events continued to run until no new themes emerged and data appeared to be saturated.

Thirdly, a short online survey with open-ended questions focusing on gaps, challenges, innovations and good practice in urgent and emergency care was administered for stakeholder groups that were not adequately represented at the events in order to capture the experiences of all stakeholders (Additional files 2, 3, 4).

The fourth data source was a process mapping activity in urgent and emergency care contexts that aimed to explore and test the consistency of themes generated from stakeholder events and gain deeper understanding of gaps and pinch points identified. The method involved discussing processes and competences with leads from 14 clinical settings where upstream or downstream pinch-points are likely to occur and then compile high level process and ‘swim lane’ maps to visually illustrate the interdependence and contribution of different contexts to the urgent and emergency care system. Swim lanes organized activities into groups based on who is responsible for the different steps within the urgent and emergency care delivery system. These provided in-depth perspectives about duplication and waste; what worked well and prompted debate on the structure of an effective urgent and emergency care system (Additional file 5).

### Participants

Participants in the study were urgent and emergency care stakeholders including representatives of service user groups. The 102 participants that attended stakeholder events organized across the region included representatives from primary/ community services (13), accident and emergency departments (*n* = 17), acute services (*n* = 10), ambulatory care (*n* = 13), residential and nursing homes (*n* = 6), ambulance services (*n* = 4), clinical commissioning groups (*n* = 3), service user groups (*n* = 16), voluntary sector workers (*n* = 3), social services and enablement services (*n* = 6) and Higher Education Institutes (*n* = 11).

Forty-eight respondents completed the online survey that targeted underrepresented groups at the stakeholder events. These included general practitioners (*n* = 8), community and hospital pharmacists (*n* = 1), specialists and allied health professionals (*n* = 30), Ambulance staff/ paramedics (*n* = 1), public/patient groups (*n* = 2) and residential and nursing homes (*n* = 6).

The process mapping activity involved in-depth interviews with leads in each of the 14 contexts identified namely: integrated discharge teams; care homes; ambulatory care units; accident and emergency departments; intermediate care teams and community beds; social services and enablement services; ambulance services; National Health Service (NHS)111 urgent care telephone advice service; hospices; minor injuries units; mental health services; rapid response teams; integrated care24; and hospital at home.

The multiple data sources facilitated triangulation, enhanced the validity and reliability of the case study and provided holistic understanding of existing challenges in the urgent and emergency service and workforce.

### Analysis

Primary data analysis involved collaborative thematic analysis with participants at each stakeholder event to empower participants to co-create a shared purpose [28]. Collaborative data analysis is the process in which stakeholders jointly focus and have dialogue among themselves about a shared body of data to produce an agreed interpretation [33]. During analysis, participants were encouraged to apply an appreciative inquiry approach to gaps and pinch points they identified about the current urgent and emergency care service [34]. This approach facilitated stakeholders to translate constraints into enabling factors through building on shared understanding of what works well in providing care that is safe, person-centered and effective.

The research team used a similar process to generate themes from the literature review, qualitative online survey and process mapping activity. Themes from each of the four datasets were maintained separately during primary level data analysis to enable cross data consistency checks [29].

The research team completed secondary level data analysis by triangulating themes from all datasets and synthesizing them in relation to broad system components [26, 27]. The data triangulation process focused on examining patterns and variations in themes emerging from all datasets. Each dataset yielded a number of themes, consistent across all sources but overarching themes for system enablers, specific workforce enablers and whole systems outcomes were resolved based on the process mapping gap analysis matrix, which provided a holistic picture of urgent and emergency care delivery covering 14 different settings.

The data analysis process entailed distinguishing characteristics of a whole system approach to urgent and emergency care and developing a representative framework of system and workforce development enablers and outcomes. Data were consciously organized using systems assumptions, into enablers (inputs) and desired outputs for whole systems urgent and emergency care delivery [26]. The synthesis for system enablers sought to address the research questions about how we can solve the current workforce crisis in emergency departments creatively to promote sustainable transformational change while specific workforce enablers aimed to respond to what the workforce of the future would look like. Additional file 6: Figure S1 illustrates the different levels of data analysis and the influence of themes from datasets on other data collection methods.

## Results

Themes from the miracle question about an ideal effective integrated urgent and emergency care system yielded the criteria for whole systems urgent and emergency care. Participants characterized an urgent and emergency care whole system as one that is safe, sustainable and person-centered; based on best evidence and practice standards; integrates health and social care; focuses on quality and safety rather than targets; is tailored to meet needs in the local population; and involves interdependent partners working together towards the same purpose. Additional file 7: Figure S2 shows the framework we generated deductively using systems assumptions [27] to achieve whole systems urgent and emergency care.

Gaps and challenges identified in the current service informed system and specific workforce enablers, building on what works and envisioning what is required for whole systems urgent and emergency care delivery. Three overarching themes prominent in the process mapping gap analysis matrix emerged from all datasets:

- fragmented working without clinical systems leadership causing duplication and waste;
- a lack of any integrating competence framework to enable staff recruitment, development and retention across all contributing partners; and
- a lack of team approach to the competences needed to cope with urgent and emergency care demands across the health economy.

We hypothesized that whole system enablers identified across datasets have implications for workforce development at every level ranging from provider-user interfaces; career development across urgent and emergency care; systems leadership; human resource management through to infrastructure development; public information systems, and commissioning.

Participants perceived commissioners to be the gatekeepers for a common strategy that models integrated working at the commissioning level and works to dismantle barriers that drive silos across the system particularly around the use of budgets and information in acute and primary care settings. One urgent care clinician commented:

*There is a lack of integrated pathways and too many clinical commissioning groups to work with.*

Additional file 7: Figure S2 outlines the overarching areas for system and specific workforce enablers and details of system enablers are presented in Additional file 8: Table S1.

Core challenges across the whole health economy were identified as both recruitment and the retention of staff and that these need to be addressed in a joined up way through system and specific workforce enablers. For example, a lead for a community healthcare team stated:

*Recruitment has been a real problem for our trust. staff are moving away from the trust towards general practice. Staff need to have acute experience and know how the systems and processes work in both the community and hospital setting. I often feel that I could facilitate early discharge if a discussion was held between ourselves and the ward staff.*

### Specific workforce enablers for a whole urgent and emergency care system

Three specific workforce enablers emerged as most significant not only for achieving a whole system approach to workforce transformation, but also for addressing issues pertaining to staff recruitment and retention. These were: i) clinical systems leadership, ii) an integrated career and competence framework and iii) facilitators of work based learning.

#### Clinical systems leadership for a whole systems urgent and emergency care

Secondary analysis of data overwhelmingly indicated the need for a much stronger focus on leadership with less emphasis on management. Themes from all datasets denoted that clinical systems leadership would complement leadership for commissioning urgent and emergency care services. This is reflected in statements made by some of the participants:

*“Integrate experienced clinicians into the system. Management needs to be much better in an integrated system. Currently ridiculously fragmented” (General Practitioner).*

*At the moment there are many senior managers and I think their role and expectations should be reviewed. There always seems to be many meetings/discussions but there are few actions generated and these are not adhered to in a timely manner. There needs to be a greater emphasis on clinical skills and practice development so that new services/skills can be explored and supported in practice (emergency care nurse).*

Clinical systems leadership is a concept we assigned to the leadership approach that drives integration across boundaries based on specialized clinical credibility working with shared purposes to break down silos and deliver person-centered, safe and effective care with continuity. Clinical systems leadership was linked to the ability to draw on expertise in a number of different areas to enable contributing partners to work together towards a shared purpose and to create a culture that values and retains staff. The required skill identified in the data encompasses: clinical expertise and credibility for a specific client group; consultancy functions that share expertise within the wider system; leadership for culture change; developing, improving and evaluating person centered care; and creating a learning culture that uses the workplace as the main resource for learning.

#### An integrated career and competence framework

Cross data analyses suggested that a single integrated career and competence framework would enable staff recruitment and retention and also empower staff to know how the systems and processes work in both the community and hospital settings. One emergency care doctor emphasized:

*There is a need to think productively, are the right resources in place, clear roles and responsibilities. Identify what skills and jobs could be done by administrative or healthcare support workers rather than removing a nurse or qualified healthcare professional from hands on care.*

Assess, Treat and SORT (support discharge, organize admission, refer and/ or transfer) constituted the single integrated career and competence framework we developed outlining key competences performed in any context by interdependent partners. Themes relating to competences required for current and future integrated urgent and emergency care from each of the datasets were triangulated to identify the key outcome competences. That is, what staff would be expected to do in the workplace underpinned by essential knowledge and understanding. We created three categories for the key competences to reflect the person’s journey through the urgent and emergency care pathway.

- Assess (alert to the need for action or assess people for urgent or emergency care within different contexts)
- Treat (treat people appropriately and promptly for their urgent and emergency care needs within different contexts)
- SORT (support discharge, organize admission, refer, transfer (SORT) people appropriately within or across the system and its different contexts in a timely way)

The key competences were mapped against national competence frameworks identified in the literature for professional groups working across the urgent and emergency care system to validate the themes generated for the single integrated career and competence framework. Additional file 9: Figure S3 outlines the single integrated career and competence framework for a whole systems urgent and emergency care workforce.

A multidisciplinary career and competence framework for urgent and emergency care demonstrates whole systems working in managing the patient pathway and experience in any context - promoting an interdisciplinary team approach underpinned by shared risk and integrated information and finance systems.

Case example 1 illustrates how a learning disabilities consultant undertaking a program to develop expertise in the functions of clinical systems leadership (aspiring clinical systems leader) works collaboratively across boundaries to ensure that a person with learning disabilities and complex needs receives appropriate care and avoids hospital admission. This case example also illustrates effective use of resources in place, and attributes of systems leadership with a focus on person centered, safe and effective care. Case example 1 was an account of the participant’s experience with the service shared at one of the stakeholder events.

### Case example 1

*A nurse working with people with learning disabilities in the community and an aspiring clinical systems leader at the hospital have been corresponding for two weeks about a person with a learning disability and an autistic spectrum condition. The person had recently been discharged from hospital to a residential care home having had a fall and a fractured ankle. The community nurse contacted the hospital about the individual’s loss of skills and mobility over the last four months along with disturbances in behavior that did not appear to be mental health related. A closer review of the records indicated that the individual had experienced two admissions and three visits to the emergency department not quite triggering the learning disability repeated admission pathway (people with learning disabilities admitted through the accident and emergency department three times or more). Diagnostics also suggested a possible malignancy. The aspiring clinical systems leader linked the community nurse with an orthopedic consultant and the general practitioner via email, encouraging coordinated discussion about the individual, which led to swift conclusion regarding the possible diagnosis of cancer and further discussions about interventions for behavior problems and loss of skills. The individual did not require another emergency admission due in part to the collaborative practice across several organizational boundaries and the individual’s care being coordinated effectively.*

Case example 2 illustrates how the single career and competence framework can be used in everyday practice for diverse contexts and roles to facilitate work based learning and career development. Case example 2 was developed by the research team to illustrate how the Assess, Treat and SORT can be used to support career development.

### Case example 2

*A community nurse working part of an integrated discharge team plays a vital role in preventing unnecessary hospital admissions by liaising with others to make sure that older frail people receive the care and treatment they require in a home setting. For example, pain management, treatment for urinary tract infections and end of life care planning, taking into account the individuals wish about their preferred place of death.*

*Formerly an emergency care nurse, the community nurse in question uses the integrated career and competence framework to undertake a self-assessment and submit a portfolio of evidence to a local university in order to gain academic accreditation for prior learning and development in the workplace. The community nurse is able to pursue a blended Master of Science advanced practice program at the university tailored to development needs identified. By demonstrating advanced skills in clinical assessment, history taking and decision making in addition to a prescribing qualification, the community nurse gains 60 academic credits towards accreditation for prior experiential learning at advanced practitioner level.*

#### Facilitators of work based learning

Facilitators of work based learning emerged as an essential enabler for supporting the urgent and emergency care workforce competence and career development while using the workplace as the main resource for learning and development. Evidence from the data suggested that facilitating learning in the workplace would enable role clarity and a team approach to the competences needed for urgent and emergency care demands across the various contexts. A community matron working with people with long term conditions observed:

*Teams from the different organizations involved are often very tribal in their behaviors, with a low level of trust. People often do not understand their colleague’s role. This is counterproductive and negatively impacts on team working. The patient experiences disjointed care. Suggest more joint training across organizations and multidisciplinary events to build trust and understanding.*

Workplace mentors and supervisors that facilitate learning, development and improvement in a holistic way embrace the integration remit across the system and meet the learning needs of multi-disciplinary teams aligned to the single integrated career and competence framework. Participants evoked that this can be achieved through rotation opportunities which enable staff to become familiar with the whole system, and the talents and contributions of interdependent partners.

## Discussion

Whole systems approaches to tackle overcrowding and promote collaborative working to improve people’s experience and health outcomes are more compelling in an era of increased demand and finite budgets. The objective of this study was to identify workforce enablers for achieving whole systems urgent and emergency care. Aligning workforce planning and development with population healthcare needs is crucial but complex, especially in light of changing needs in an aging population that present with a complex range of mental and physical morbidities [35, 36]. However, integrating change across structures, processes and patterns facilitates the achievement of transformational change in a complex system [10]. Participants in our study comprised a range of stakeholders from different settings of urgent and emergency care including representatives of service user groups to mirror collaborative ways of working that can achieve a shared purpose and draw on the users’ perspectives as the organizing principle of service delivery [37]. Results suggest that whole system working is achieved through both systemic and workforce enablers which may receive less attention in health care redesign other than essential structural and process changes like standard setting or information and financial infrastructures which on their own, do not deliver improvements in quality and performance [9].

The most consistent finding was the need for strong systems leadership capable of enabling the creative reshaping of urgent and emergency care services while coping with change. Systems leadership is often presumed to be the remit of commissioners of healthcare services [38] however the need for clinical systems leadership demonstrated in this study is a new concept linked to expertise in a number of functions surrounding culture change and clinical credibility in the workplace. Clinical systems leadership aims to work with behavioral norms, develop common values and a shared purpose that positively impact on workplace cultures, ways of working, team work, staff wellbeing and satisfaction, as well as patients’ experience and outcomes, and efficient use of resources [30, 39]. These findings have direct relevance to workforce strategies addressing existing discrepancies between ideas of how to achieve this and real investment in developing systems leaders [40].

Results of this study indicate that system redesign and successful implementation is facilitated through enabling the workforce to acquire competences needed to cope with complexity and changing models of care [36]. In addition to retaining and valuing the existing urgent and emergency care workforce, the task encompasses developing competence across different contexts comprising the patient pathway. Competence can be developed and strengthened through a single integrated career and competence framework supported by clinical systems leaders for different client groups working together across the system. This study showed that an integrated career and competence framework that is not role dependent provides a more integrated and flexible career pathway to facilitate progression both vertically and horizontally; identify skills gaps in practice; inform learning and development needs, and aid supervision and mentorship in different contexts. Competence need not be vested in one person, profession or staff group, but supported by partners in different contexts embracing collective responsibility and capability that supports transformational change [36].

Workplace facilitation spans a number of purposes including learning, development, improvement, inquiry, knowledge translation and innovation [41]. Results of this study imply that a whole systems approach to skilled facilitation of learning in the workplace brings together multiple programs for learning which, in isolation may exert additional pressure on staff who already feel ‘time poor’ in the presence of increasing demand for patient care [42]. Integrated work based learning offers rotation opportunities that promote the development of holistic and systemic competence, and enables practitioners to acquire the knowledge and know how to succeed in their roles and to demonstrate behaviors required to effectively manage situations they are likely to encounter in various urgent and emergency care settings.

### Strengths and limitations

This study illuminates whole systems working which involves integrating all system elements including structures, processes and patterns that drive thinking and behavior in the workplace to achieve transformational change.

The collaborative and inclusive approach used the study demonstrates a partnership drawing on providers’ and users’ experiences to achieve shared direction and valued outcomes. This approach is most likely to obtain and sustain positive change in the delivery of care.

Nevertheless, it is probable that stakeholder events may have inhibited freedom of expression as opposed to user only groups. While this resulted in powerful case studies to illustrate an ideal system, the same drawback may have also influenced discussions with junior staff involved in delivering urgent and emergency care.

## Conclusion

In this study, participants agreed that whole systems urgent and emergency care allows for the design and implementation of care delivery models that meet complexity of population healthcare needs, reduce duplication and waste and improve healthcare outcomes and patients’ experiences. For this to be achieved emphasis needs to be placed on holistic changes in structures, processes and patterns of the urgent and emergency care system. Often overlooked, patterns that drive the thinking and behavior in the workplace directly impact on staff recruitment and retention and the overall effectiveness of the organization. These also need to be attended to for transformational change to be achieved and sustained. We recommend further work to refine and validate the integrated career and competence framework by testing competences against all key roles in different contexts across interdependent partners contributing to the system; and to identify standards for integrated facilitation in the workplace to grow the capacity of work based facilitators and ensure consistency in quality and effectiveness of this role.

## Abbreviations

NHS, National Health Service; SORT, support discharge, organize admission, refer and/ or transfer; UK, United Kingdom

## Additional files

Additional file 1: Case stories. (DOCX 23 kb) Additional file 2: Questionnaire for team leads, allied health professionals and specialist nurses. (DOCX 20 kb) Additionla file 3: Questionnaire for general practitioners, pharmacists, residential and nursing homes staff and paramedics and ambulance staff. (DOCX 24 kb) Additional file 4: Questionnaire for public/patients. (DOCX 20 kb) Additional file 5: Process and gap analysis matrix. (DOC 89 kb) Additional file 6: Figure S1. Levels of data analysis. (DOCX 50 kb) Additional file 7: Figure S2. Framework for achieving whole systems urgent and emergency care. (DOCX 34 kb) Additional file 8: Table S1. System enablers for a whole system approach to urgent and emergency care delivery. (DOC 32 kb) Additional file 9: Figure S3. Integrated career and competence framework for whole systems urgent and emergency care workforce. (DOCX 26 kb)
