# Supplementary material for: Using systems thinking to identify workforce enablers for a whole systems approach to urgent and emergency care delivery: a multiple case study
Source: BMC Health Serv Res. 2016 Aug 9;16:368. doi: 10.1186/s12913-016-1616-y (PMC4979146; doi:10.1186/s12913-016-1616-y)
Supplement: Additional file 1: — Case stories. (DOCX 23 kb) [file 12913_2016_1616_MOESM1_ESM.docx]

**CASE STORIES**

**Story 1: Residential home, GP, Hospital & Pharmacist**

A relative suffers from severe dementia and lives in a residential home without nursing care. In May this year, my relative presented with seizures requiring admission to hospital twice within the same month. During the second admission my relative had to be restrained in a chair on the ward to prevent wandering and on discharge spent 7 hours in the discharge lounge waiting for an ambulance, this caused significant distress to the patient. My relative was discharged on a low dose of an anti-epileptic drug.

I was concerned that the dose would not be titrated to a therapeutic dose, resulting in the patient requiring another admission to hospital. Following a discussion with the carers at the home to determine what level of care they could provide, I phoned the general practitioner (GP) to agree a management plan. The GP agreed to titrate the dose of the anti-epileptic drug to the recommended maintenance dose depending on the patient’s tolerability to the side-effects e.g. behavioural disturbances and drowsiness which could precipitate falls. The GP was happy to treat my relative at the home to avoid an unnecessary hospital admission provided that the fits had resulted in no harm or not developed a more sinister pattern.

The staff in the home were positive about this arrangement as they had concerns about the standard of care for dementia patients in an acute setting and felt they could provide better care as they were more familiar with the patient’s needs.

It has been 7 months since my relative’s last admission to hospital, has not suffered from any subsequent fits and hence had no further hospital admissions.

The GP treatment plan was key in addressing the management of the fits and keeping the person with dementia at the care home where their needs could be better met and they would be well cared for. Also support of the care home staff was important but their satisfaction too was a bonus. Without the treatment plan there was the danger of under treatment and the fits recurring. In addition, the normal policy of the carers at the home was to contact the hospital in the first instance if the resident should deteriorate. I wanted the carers to phone the GP first for advice as the GP was willing for this to happen and also knew the patient well.

**Story 2: Ambulatory Care**

A 52-year-old was referred the same day directly to Hot Ambulatory from the GP, removing the need for the person to be seen either in A&E or wait for an outpatient appointment.

The GP discussed the clinical presentation and the person’s suitability for Emergency Ambulatory Care with an acute physician – the person had developed lumps in the neck. The person was able to drive to the hospital and did not require an ambulance.

On arrival at the ambulatory unit, the person was booked in by the receptionist, and shown to a trolley space for examination and assessment by the acute physician. Blood samples were taken and on the basis of these results, the person was booked in the following day for Ultrasound and CT Contrast. The person was discharged from the ambulatory unit at 17.00 and asked to return for review the following afternoon immediately after the Ultrasound and CT.

The person attended as planned for the results of the scans, which showed abnormalities that unfortunately had spread to the lungs. Whilst in the unit, the person was then booked for a CT guided biopsy on the following day through the Ambulatory Unit. On discharge the person was given an appointment for the following week on 24/10 for follow-up bloods to be discussed at the Lung MDM.

The system allowed for a seamless patient journey from attendance at the GP surgery directly through to the Ambulatory enabling the person to receive the right care, at the right time and in the right place – putting the individual at the centre of care.

***Researcher’s Feedback***

*An example of swift, seamless care delivered through Hot Ambulatory. It exemplifies the positives of integrated working and ensuring that the care we provide is patient centred and the patient’s journey is seamless.*

*We felt that this was the future direction of acute care and would fit nicely with the feedback form the stakeholder analysis that we a have undertaken, in reference to having the right person, with the right skills, in the right place at the right time and that the patients journey should be one stop.*

**Story 3 111 Call Handler/ambulance**

A third sector worker talked about the experience of using 111. The third sector worker phoned 111 because a relative wasn't feeling very well and was therefore concerned.

The third sector worker had to answer 'loads' of questions which was appreciated as the call handler was doing a job but nevertheless the caller still felt frustrated. Anyway the caller went through the process, spoke to the relevant people and thought that was it! That was until an ambulance turned up several minutes later to see the patient. The third sector worker hadn't been told this, didn't want an ambulance and just felt it was a waste of resources.

“I know it doesn't appear to be an exciting story but I do think it is pertinent that it reflects the need for the call handlers to have the appropriate skill set to be able to make clinical judgements rather than follow a predesignated algorithm” [ third sector worker].

**Story 4 Hospital Person centred care – stroke patient**

One individual shared experience about the care received when the partner had a stroke. The person dialled 999 on realisation of what was happening and the ambulance was dispatched and responded very quickly. The partner was taken to a local emergency department, where again the patient was treated promptly and received the correct treatment.

To that point the couple had had a very positive experience, which unfortunately changed when this person’s partner was then admitted as an inpatient. Here the experience was more negative, in that the communication was poor and the partner’s progress was not explained to them. This resulted in them both desperate for her to go home.

Fortunately, the patient recovered from the stroke but they wished that the initial care experienced was consistent though out the whole of the patient’s journey.

**Story 5: A&E - Children**

My child suffered a head injury after a fall while playing. I called 111 for help when my child started vomiting and was very drowsy. After 20 minutes on the phone speaking to a non-clinical call handler, paramedics were sent out to see and possibly treat. The paramedics were fantastic!

They carried out all tests they could do at the time but were worried that my little one was quite drowsy. They decided to take my child to a local district general hospital for further assessment. The handover took about 15 minutes. My child and I sat in the waiting room with older people, some drunk young men, a few other children with their parents, people who suffered open wound injuries, young vulgar couples......... people from all walks of life.

The waiting time indicated on the board was 30 minutes but after 1½ hours of waiting it was changed to 1 hour. Did that mean we had to wait another hour? Everyone we met in the waiting room was attended to and left. When five people who had arrived later left, my child asked if anybody could still remember the child’s head hurt so much. I inquired at reception and was informed that my child was to be treated in the major and not minor injuries unit.

Following further inquiries I was informed that we had to wait for three more people to be treated. We were then offered a trolley. 40 minutes later my child was attended to. Lucky enough we were discharged to go home. The doctor said that my child would be given some Calpol to relieve the pain experienced at the time but the doctor did not specify who was to do it. I asked one of the Emergency Department nurses who snapped back that they didn't have any Calpol to give people. Another staff nearby asked me, "do you know where Tesco is? I could have taken my child to Tesco if that was the most appropriate place to seek health care for a head injury I thought to myself. I bit my tongue but at the reception there is a note that reads 'if in pain ask for relief' and another that instructs you not to take any medication before your treatment. Patients are already confused by the degree of inconsistency and incompetency sometimes present in many A & E departments.

We pay so much in taxes every month and so I am wondering is this the best quality of urgent care the NHS can give? Are there any standards and/or competences required for paediatric health care? I think it's a disgrace.

**Story 6. Aspiring clinical systems leader**

'During the last two weeks the community learning disability nurse and I have been in correspondence regarding a person with a severe learning disability and autistic spectrum condition who had recently been discharged from hospital having had a fall and fractured ankle to a residential care home. The community nurse contacted me regarding the individuals loss of skills and mobility over the last four months along with disturbances in behaviour which did not appear to be mental health related. On closer review of the records it was noted that the individual had experienced 2 admissions and 3 visits to A&E not quite triggering the Learning Disability Repeated admission pathway, there was also reference to diagnostics suggesting a malignancy.

We were able to link up the community nurse with the orthopaedic consultant and the GP via email, encouraging coordinated discussion regarding the individual, swiftly discounting the queried diagnosis of cancer, but there remained concerns regarding the behavioural changes and loss of skills. There were further discussions about how to support the individual with behaviour problems and loss of skill.

The individual did not require emergency admission over the weekend, due in part to the collaborative practice across several organisational boundaries and the individual’s care being co-ordinated effectively.'

**Story 7. Community nursing practitioner demonstrating how a joined up career and competence framework could be used**

A community nursing practitioner working part of an integrated discharge team plays a vital role in preventing unnecessary hospital admissions by liaising with others to make sure that older frail people receive the care and treatment they require in a home setting. For example pain management, treatment for urinary tract infections and end of life care planning, taking into account the individuals wishes about their preferred place of death.

Formerly an emergency nurse practitioner, the community practitioner in question uses the integrated career and competence framework to undertake a self-assessment and submit a portfolio of evidence to a local university in order to gain academic accreditation for prior learning and development in the workplace. The practitioner is able to pursue a blended MSc advanced practice program at the university tailored to development needs identified. By demonstrating advanced skills in clinical assessment, history taking and decision making in addition to a prescribing qualification, the practitioner gains 60 academic credits towards accreditation for prior experiential learning at advanced practitioner level.

**Story 8. User perspectives on urgent and emergency care**

**“**People with Parkinson's (PwP) being taken to A&E, or minor injuries, on their own may have all their meds with them but may not have a 'carer', and this can result in them being completely ignored for 5 hours or more during which time they should have had medication, food, water and the chance to use a toilet (bottle or equivalent). Even once the patient is seen the staff start 'ordering' medication rather than using what has come with the patient, so the patient becomes much worse (it is according to the books dangerous to stop Parkinson meds suddenly) and stays in hospital for ages even if there was no good reason for their presence there in the first place. A&E doesn’t (for good reason) serve food or drink or do 'care'(meds and 'bottles') and this makes it a very dangerous place for PwP to be! They could save the hospital a fortune by a) not taking people with Parkinson's unless there is a VERY good and obvious reason that will get treated immediately; b) highlighting Parkinson's at the Triage stage and dealing with it in a timely fashion and c) using medication that comes with a patient (with a little common sense that is sadly lacking in any situation where people might get sued!) I guess you all get the message; will they?” (Patient)

**Story 9. Interdependent system partners are reliant on one another to ensure seamless, patient centred care**.

A patient with end stage bronchiectasis had home O_2_ and nebulisers set up and a good care package. Through the end stage of the patient’s life, the patient developed a chest infection that required close monitoring and complex long term IV antibiotic therapy. The hospital at home service worked with the patient, the patient’s family, physiotherapy and the community matrons to provide a unique package of care that enabled the patient to stay at home for the last 6 months of the patient’s life whilst still receiving close monitoring, the complex IV therapy and good communication links direct to the patient’s consultant.

**Story 10. Collaborative approach to maternity telephone triage**

An excellent example of partnership working comes from the southern coast of England, where the maternity services and ambulance service run a telephone triage service for pregnant women. Midwives offer this service based within the ambulance control centre. Partnership benefits include immediate access to ambulance when required, sharing of expertise and better understanding of partner organisation. In addition to calls coming through for the maternity triage service, midwives are able to offer advice and support when pregnant women dial 999 or 111. Women benefit from the midwives being able to focus fully on their needs and savings have been made by ensuring appropriate use of the ambulance service.
