# Supplementary material for: Using systems thinking to identify workforce enablers for a whole systems approach to urgent and emergency care delivery: a multiple case study
Source: BMC Health Serv Res. 2016 Aug 9;16:368. doi: 10.1186/s12913-016-1616-y (PMC4979146; doi:10.1186/s12913-016-1616-y)
Supplement: Additional file 2: — Questionnaire for team leads, allied health professionals and specialist nurses. (DOCX 20 kb) [file 12913_2016_1616_MOESM2_ESM.docx]

**Developing the Future Urgent and Emergency Care Workforce**

**Questionnaire for Team Leads and Specialist Nurses/Allied Health Professionals Caring for People with Long Term Conditions**

1. What factors and/ or skills are currently lacking and therefore hinder the provision of joined up and smooth running urgent and emergency care to patients with long term conditions when they need it?
2. What skills (clinical, administrative, managerial) are needed for future provision of a joined up, smooth running and lasting urgent and emergency care service?
3. What new ideas are available to practice that enable patients with long term conditions to experience a joined up and smooth running urgent and emergency care service?
4. What new ideas would you like to see introduced to enable patients with long term conditions to experience a joined up and smooth running urgent and emergency care service?
5. What other comments would you like to make about how a joined up and smooth running urgent and emergency care service could be provided to patients with long term condition?

**Thank you for taking part in this survey.**
