# Supplementary material for: Using systems thinking to identify workforce enablers for a whole systems approach to urgent and emergency care delivery: a multiple case study
Source: BMC Health Serv Res. 2016 Aug 9;16:368. doi: 10.1186/s12913-016-1616-y (PMC4979146; doi:10.1186/s12913-016-1616-y)
Supplement: Additionla file 3: — Questionnaire for general practitioners, pharmacists, residential and nursing homes staff and paramedics and ambulance staff. (DOCX 24 kb) [file 12913_2016_1616_MOESM3_ESM.docx]

**Developing the Future Urgent and Emergency Care Workforce**

**Questionnaire for General Practitioners, Pharmacist, Ambulance Staff/ Paramedics and Residential/ Nursing Home Staff**

1. Please indicate the professional group you represent.

General Practitioners

Community & hospital pharmacist

Ambulance Staff & Paramedics

Residential & Nursing home

Other (please specify)

1. What factors/competences/skills (clinical, administrative, managerial) are currently lacking, therefore hindering service provision?
2. What are the competences/skills (clinical, administrative, managerial) needed for future provision to be integrated, seamless and sustainable?
3. What innovations is your service contributing to an integrated seamless urgent and emergency care service?
4. What innovations would you like to see introduced to enable an integrated seamless urgent and emergency care service?
5. What other comments would you like to make about the workforce requirements needed to deliver integrated seamless urgent and emergency care?

**Thank you for taking part in this survey.**
