# Supplementary material for: Using systems thinking to identify workforce enablers for a whole systems approach to urgent and emergency care delivery: a multiple case study
Source: BMC Health Serv Res. 2016 Aug 9;16:368. doi: 10.1186/s12913-016-1616-y (PMC4979146; doi:10.1186/s12913-016-1616-y)
Supplement: Additional file 4: — Questionnaire for public/patients. (DOCX 20 kb) [file 12913_2016_1616_MOESM4_ESM.docx]

**Developing the Future Urgent and Emergency Care Workforce**

**Questionnaire for the Public/ Patients**

1. What factors and/ or staff skills are missing that hinder the provision of joined up and smooth running urgent and emergency care when the public needs it?
2. What are the skills (clinical, administrative & managerial) needed for a future integrated, smooth running and lasting service?
3. What new service ideas are you aware of that enable the public to experience a joined up and smooth running urgent and emergency service care?
4. What new ideas would you like to see introduced to enable patients/ public to experience an integrated and smooth running urgent and emergency care service within the limited funds?
5. What other comments would you make about how a joined up and smooth running urgent and emergency care service could be provided to the public?

**Thank you for taking part in this survey.**
