# Supplementary material for: Using systems thinking to identify workforce enablers for a whole systems approach to urgent and emergency care delivery: a multiple case study
Source: BMC Health Serv Res. 2016 Aug 9;16:368. doi: 10.1186/s12913-016-1616-y (PMC4979146; doi:10.1186/s12913-016-1616-y)
Supplement: Additional file 5: — Process and gap analysis matrix. (DOC 89 kb) [file 12913_2016_1616_MOESM5_ESM.doc]

**Process & Gap Analysis Matrix**

| 1. Stakeholder | 2. Current Issues/Challenges | 3. Good practice/working well | 4. Miracle Questions | 5.Future Skills/Competences |
| --- | --- | --- | --- | --- |
| A&E | - A lot of the current staff do not have the required competences to cope with the demands - Huge variance in skills within a staffing band - Difficult to keep track of competences of staff that they have undertaken or they are used - E-rostering dictates what competences you have on shift as not all banded staff have the same. Ie ENP/ Paed nursing and during annual leave - Recruitment issues to fill vacancies - If nursing moves up a grade leaves a gap below which cannot be filled with required skill sets - Backfilling an issue - Reliance on locums - Poor performance not management through - Spilt between Dr v nursing – lack of team working & accountability - ENPs working in isolation to rest of A&E Dept - Inappropriate patients being sent in or self-referring - Not enough access to shared records - Lots of investigations mean a wait for the patient – are they the correct ones being requested? Does the patient need all of these? - Repeat attenders and readmissions not dealt with proactively - case conference - Access to beds - Patient transport - Triage – streaming by admin staff - Not enough Paeds nurses - Difficulties in achieving 4 hour standard resulting in patients sometimes being admitted if investigations not done/completed in A&E setting | - Transferrable skills with some good skill sets for trained staff at band 5 & 6 - Some parts of roles are good, ie band 7 skills good leadership in clinical resus event but not in management - Attractive roles for nurse managers/leaders but can leave gaps with lesser skilled band 6’s left below. - Embedded pathways give a good patient experience with staff understanding their involvement/role - GP at Front Door – but could see more (Commissioning is a blocker) - Mental Health Liaison located within A&E 24/7 - SEAU – streamlines patients to correct surgical specialty - Pulling patients straight to HOT ambulatory - MINT nurses - Dedicated portering services | - Sharing of information between providers and referrers - Clear system of how to discharge/refer to appropriate services for patient – clear signposting - Electronic cascard system which links to the PAS system - Electronic SECAMB system accessible to A&E - Staff should be able to work across the Emergency Floor - OOH work with OOH GP service/IC24 to stream patients away from A&E - Working hand in hand with other providers such as primary care /Community/ mental health/Social Services - Ability to appropriate workforce plan rather than to fire fight against demand - Ability to effectively refer suitable patients for tele-health for management of LTCs | - Method of showing staff competences and booking shifts appropriately - ICPs – standardised with prescribed care plans - Attributes of management training for higher banded staff to lead - Reflective practice to look at how things went and how to learn/improve - Named nurse to pull patients through the system - TV skills or specialised nurses at front end - Project management skills - Clear role for Physicians Assistant - Incorporating ENP/ANP role more into A&E/Minors - Leadership from managers to give vision - Specialised skills to enhance A&E skills such as Paeds, TV. - Skills and competences aligned to other specialised areas such as Resus & ITU re intubation - All nursing staff able to cannulate - Recognising deteriorating patient - Dementia toolkit |
| Secamb | - A lot of unnecessary calls - Unable to prescribe - Not enough Paramedic Practitioners - Too many pathways for paramedics to look at in order to signpost effectively at the scene - IT not shared effectively - Competencies and pay bandings do match Acute setting - Paperwork – is it read? Is it enough? - Very process driven and prevents free thinking - Activity increasing - Differing systems across the different areas, ie LRU for Canterbury & Ashford and not for other areas | - Able to signpost a lot of calls when received - Other stakeholders open to sharing pathways and SECAMB taking direct, ie RR, Ambulatory pilot - Paramedic Practitioners able to be first responders in cars and able to assess situation - Use of the support workers - Good leadership - Dedicated GP numbers to arrange GP appts by Paramedics at the scene, - Access to GPs at A&E/MIU to make appts/gain advice avoiding admission - Good see and treat skills diverts attendance at MIU - Able to bypass A&Es and take direct to MIUs etc - Building up the IBIS database – useful for LTC management - Less patients transferred to acute setting despite rise in total number of calls | - Organisations being able to work together - Shared ownership - One IT system able for all to access and share records and care plans - Similar processes with one DOS booklet to show pathways - Secamb practitioners paid at the level of competence to other staff – such as Trust ENPs, ANPs etc | - Prescribing - Paramedic Practitioners trained to ANP level, with more band 7 senior decision makers - Shared competency levels between organisations/consistency - Role of Emergency Practitioner in Nursing/Care Homes to be developed - Skills in identifying Sepsis and early giving of AB’s |
| 111 | - Staff turnover gives variance in quality and interpretation of process tool. - Signposting set for 15 miles – this can direct patients to the wrong areas is the zone goes over the sea, ie Sheppey - Some of the outputs are inconsistent, ie for urgent apt but the patients are not urgent when seen - Nowhere to record it patient has LTC - Wrong addresses are picked up - No links to any records, ie GP, share my care etc | - Training programme – varied and offering wide range of training media - Service more widely recognised and usage has gone up - Clinical support by floor walking GPs to assist in managing complex calls - Facility for clinical call back to the patient | - Links to electronic shared records - Promoting 111 more – untapped potential and building confidence - Improvement in how the DOS is updated and what is contained to enable operators to signpost correctly - Ability to hold onto good staff – turnover huge | - Ability to signpost more effectively - To give a service which is more person centred and not evident it is delivered from a flow chart |
| Ambulatory | - CCGs providing some services such as DVT without correct training/competences giving poor consistency - Difficulties in recruiting right level of staff, ie Acute Physicians, highly trained nursing staff - Silo working with Acute v community | - Enabler to prevent admission - New Hot Process models to treat all patients as ambulatory as proven otherwise - Amb Score - Nurse Prescribing - Quick and responsive decision making - Use of Band 4 nurses is increasing - Review clinics - Whole system pathways – linking to H@H and Community | - Ring fenced areas to enable flow of patients without impact of bed capacity issues - Resourced service to enable 7 day working for consistent service - Seamless links to Community Nursing teams - Whole systems working - Signposting via 111, LRUs - Use of Hot clinics to enable patients to return without admission - Shadow working with other providers | - Sharing of nurse led discharge in Acute setting - More IV trained Community Nurses to deliver IVants/canulate - More band 6/7 nurses to enable nurse led pathways and decision making - Physicians Assistants to fill gap between Acute Physicians and Nursing staff - Emergency Floor Doctors rotating roles into Ambulatory, CDUs and A&E including medical rotas to share knowledge and ownership |
| Care Homes | - Input by CNS staff is predominated by Quality RCAs and admission avoidance and not training - Differing levels of support given to Homes by GPs – due to locality, personality and priorities of CCG - Care Homes “labelled” by Acute setting for sending patients in - Lack of consistency around geriatrician roles in Community - Varying support with dementia residents, particularly around escorts - Varying links and accessibility to specialist areas such as Tissue Viability, RR, Enablement etc - Complex patients - Task orientated care plans which prevent staff from being person centred - How to identify the deteriorating patient and escalate appropriately - Readmissions - Levels of training, skills & motivation of staff | - Sharing of information such as Dashboards by the Acute Trusts on admission - Ability to focus on homes with high admission rates - Advanced Care Planning for some patients - CCG Operational groups – MDT focus - Medical input by Acute Geriatrician in Community - Board Rounds with MDT in Acute setting | - Communication and focused MDT Board Rounds - Consistency of service - Training packages for homes for all staff groups - Leadership and Visionary skills evident in all homes to enable quality serviced for residents - Acute Trusts to recognise importance of involving care homes in discharge planning - Reduce and breakdown barriers to enable specialties to in-reach to NH to prevent issues such as Pressure Ulcers and to RH to provide short term enablement – quality benefit to the residents. - Learn from other countries around models of looking after older generation | - Increased role of CNS to give leadership, quality and training advice to Care Homes - Give level of support to both Residential and Care Homes regardless of trained member of staff - Dementia training to all staff - Person centred skills - Agreement of skill sets for all levels of staff in care homes - Peer Review of homes by GPs and other homes - Use of ANPs and PA s to support delivery of care - To link with educational establishments to embed learning ethos and clinical supervision and retain staff - Nurse Prescribing – Community - Student nurses to play vital role as fresh pair of eyes and sharing up to date knowledge |
| Hospice | - Accuracy and reliability of referrals - Need to “eyeball” patients first - Bed availability if a patient requires and wants an urgent admission - Difficulty in recruiting senior staff, particularly in geographical areas | - Good range of options for the patient - Hospice at Home service to support patients and carers enabling patients to remain at home – run by Band 3 nurses and gives quick response - MDT approach – reduces boundaries of roles and work as a team - Patients can be seen in their own home, OPD etc gives flexibility - Highly skilled band 6/7’s to work alongside Drs - Share my care – prevents admissions and enables patient to be treated at home - 365 days a year help line for known patients to access service – patients and carers - Person centred care at all times delivered by strong MDT team ethos - Low turnover of staff | - Band 5 nurses to be part of the Hospice at Homes service to widen the number of patients that can be seen - Dovetail to Community Services - All service providers have access to Share My Care or similar - Extend helpline for all patients/carers requiring service | - Community services to work with teams to share learning around pain relief and IVs - Mini referrals to be developed to enable assessment and access to the SPA - Sharing of learning and strengthen relationships with other providers at all levels of the teams to enable appropriate referrals - 24/7 advice line with appropriate clinical knowledge to reassure & signpost – user/provider - Rotation of staff and increasing roles, ie AP will enable gaps in recruitment of current establishment |
| IDT | - Difficulties in leading and managing a multiagency team - Pull system sometimes fails due to personalities rather than roles - Split between Health and Social Care - Barriers of timescales, ie choice, time to assess cause delays in the pathway and can be used as obstacles - Person centred is not obviously priority - 7 days working gives variance of service in some of the disciplines - Capturing of what is not working - Referral process to DNs despite in-reaching - Continuing Health Care and Fast track services not covered by IDT – poor responses affecting patient wishes | - MDT team involving all stakeholders required for discharge and admission avoidance at front end of acute and with strong links and understanding of community processes/systems - Cluster working with band 5-7 nursing staff - “Pull” system in order for process to work proactively, focus on front door - Prevents admission through the different stages of the acute setting - Split of Front and back team gives clarity around focus areas - 7 day service - Access to Share My Care - Links to Community Neighbourhood teams to support transition of care - Good use of admin staff to expand boundaries - Use of voluntary sector to provide additional services such as Red Cross, Carers first – to give info to carers etc - Access to Mental health Liaison services 24/7. - All health staff able to do basic physio assessments with some able to order equipment | - Breaking down of barriers between health and social care - Communication and awareness by service users of who to contact - Shared records used by all on same system for Kent and Medway - Shared and responsive pathways across whole systems, ie DNs, Fast Track, CC - Identify and adapt discharge processes to make - Services to in-reach and be part of IDT - Continuing Care, Fast Track - Involvement in ward based MDT meetings - To be central information point for all discharge related processes and procedures for both staff and patients and relatives - Dementia specialist to be included in Front end team - Respite wards | - Leadership skills for all membership - Team working and shared purpose – values clarification - Team must be MDT with access to services/advice not part of the team - Use of PA’s to assist with EDN, drug issues and clinical input to enable discharge - Decision making ability - Development of tools for MDT - Appropriate capacity planning to enable planning of DN workload - Dementia training - Nurse prescribing - Nurse led discharge |
| IC24 | - Confidence around 111 and call handler skills - Commissioning and integration - Negativity between staff and practitioners - Other providers – continuity and recruitment/retention issues - Folkestone GP pilot 7 day working led to increase in IC24 contact - GPs expensive - Agencies competing – rates of pay plus difficult to assess competencies and quality – lack of control | - Good ANP and paramedic are vital to success - Paramedics drive themselves as opposed to Dr staff who won’t - Collaborative working with SECAMB - Lots of data available to share | - Removal of barriers - Rotational posts for staff through ED, MIU etc – evaluate to see competences - Primary care facility placed in secondary care facility, ie Isle of Sheppey - Standardised rates of pay for ANPs | - ACP and paramedic practitioners - Nurse prescribing – also linked to clinical examination - PGD’s linked to clinical assessments - National standard for advance practitioner training - Paramedic and practice nurses have different skill set but can all be called practitioners - Needs to be on NMC register - Understand what skills/competency deficits are – looking at in hours v OOH - Needs to be MDT |
| Community Matrons/Teams | - Referral process is inconsistent - Difficulties in understanding which competencies staff are supposed to have - Boundaries of which teams take which patients – linked to GPs - Capacity issues – tend to get send referrals which are not appropriate and expected to pick up - Inappropriate referrals - Block contracts can cause issues of ownership both for community and acute trusts - Trust of knowledge between professionals from other providers - Discharge information from Acute not clear or correct on occasion | - Range of banded staff from lower bands to 8A - In-reach to Acute setting - Community Matrons to form part of IDT - Use of Care Plans – creating and updating - Point of contact for many patients - Patients rely on DN visit – could attend practice? | - Seamless whole systems working with clear roles and responsibilities - Pulling patients by community matrons in-reaching diverting to other schemes such as telehealth - Sharing care plans with other stakeholders - To work with other initiatives – such as Hospital at Home, Ambulatory to form part of pathway - To include more at the front-door with their knowledge of the patient and links to GP | - Consistent competencies - Demand & Capacity and availability - Expand use of telehealth and technology - IV access - Ability for staff to accept greater range of patients. - Shadowing and sharing learning |
| SS/Enablement at Home | - Care Managers in acute and community have limited understanding of processes which impacts when covering 7/7 - Working to timelines - Communication with ward teams and patients/carers - Not documenting in Medical notes – working in isolation - Out of area patients – difficulties in resolving due to boundaries | - Social Care Discharge Coordinators integral to steam-lining acute to community - Cluster working in acute setting as part of IDT - Access to step down/social services beds - Bed Manager giving central availability feeding into IDT - More autonomy around authoring care packages and enablement - Use of KEAH for short term care packages | - Linking of whole systems removing barriers such as timescales of 72 hours to assess – moving away from task and process to person centered - To really work as an integrated team (health and social) | - Shadowing all areas of SS responsibility to give knowledge and understanding of processes & systems - Shared purpose - Leadership - Ability to move away from process and timelines to person centred approach |
| Rapid Response & ICT | - District Nurse referrals are not consistent - Referrals for DNs not always appropriate – could be seen by practice nurse - Delays with Continuing care – poor process and response. And Fast Track. - Lack of beds for CC, F/T and for general community patients - Difficult to find rehab placements - Delays in Community Hospitals discharging due to Social Services delays in setting care packages - Focus in Community Hospitals is not EDD - Bed managers in Community finish as 4 - Overlap on occasion with Acute POTS and RR. | - Band 5 and 6 nursing teams – nurse led service for RR - Good integrated working with Physio and OT rehab service - Assessments carried out by all levels of staff from band 4 – 6 - Range of services can be offered, referral to POTS, SS, Care Package, Reablement, Community Bed - Referral process open to include most patients categories - Support IDT - Provide care packages for immediate start providing have capacity - Support SS care packages providing a date is set to start - Support workers can set goals with the patient - Use CIS which SS also use to enable shared records - Have competencies around equipment, POT basic assessments | - Shared records system which all disciplines have access to - Streamlined process for CC, fast-track and Rehab beds to enable patients to be transferred to appropriate environment. - Focus on patient pathway in community - Joined up 7 day process by all agencies - Links to navigation centre/LRU to ensure correct signposting and quality service to patient - Specialised clinics to be run in community by POTS – ie Hips & Knees – led by Band 4 enhanced recovery and quicker discharge for patient - Early supported discharge for Stroke – link Acute and Community POTS | - Leadership – encouraging flow and patient pathway/experience focus - Wide range of clinical competences |
| Hospital at Home | - Referral process to be robust/consistent - Overlap between community and H@H caseload v commissioning from CCG - Not able to hand patients over to Community staff as do not have capacity or training to take on patient - Lack of IV skills in community - Limited amount of patients able to be suitable - Both Push and Pull system to H@H which is dependent on the individuals in charge or visiting the ward as opposed to automatic process - Not all specialties are included - Some Consultant resistance to referring to a virtual ward - Geography of where patients are located could make the service inefficient - Pressure to take unsuitable patients due to bed pressures | - Patient experience excellent - Outcomes good - LOS not increased - HAI rate is less if H@H take patients - Good decision making and clinical skills in band 5/6 staff - Virtual MDT ward rounds ensure whole clinical team approach - Committed Team approach - Embedded with Surgical areas | - Joined up working with Community Services to enable seamless quality service - Service to be expanded to take band 3 nursing staff and above with POTS to enhance the numbers of patients that can be taken and the care that can be delivered - Closer working with front end, ie A&E, Ambulatory to enable care to be given closer to home - Working with all specialties to enable the benefits of the service to be available - Work as part of the IDT - Engagement with Community Pharmacy teams - Links/working with Hospice @ Home team | - IV skills in community - Understanding of services by ward teams to enable appropriate referrals to take place. - Signposting from A&E & Ambulatory to deliver specialised nursing advice - Leadership skills to evidence benefits and further develop service - Links/provision of tele-health and specialised nursing teams - Awareness of other services etc which could help patients be further managed at home, |
| MIUs | - Not all diagnostics available - Different opening times may cause patients to go to Acute sites - Signposting sometimes means patients are seen and then sent to Acute A&E – delays in patient treatment - Sometimes feeling of isolation due to working in small teams | - SECAMB now take patients directly to some MIU - Led by ENPs – good all round skills and knowledge - Matron overseeing - Use of telemedicine - Nurse led discharge - Nurse led prescribing – some PGDs - Team working approach - Leadership skills - Can re-attend for some dressings but should go to practice nurse - Good signposting skills and links to GP practices | - Links to GPs/GPs working out of MIUs would increase the number of patients being seen and support nursing staff - Deliver Ambulatory pathways - Access share my care or electronic records, particularly around LTC and repeat attenders. - Links to community teams and CCGs to address repeat attenders for ACPs - Could take more if enough staffing and Radiography/Sonographers available to support - Extend SECAMB to take more patients to MIUs | - ANPs and PA’s would increase the types and numbers of patients which could be seen - Diagnostic training to nursing staff, ie ultrasound - Basic Physio assessment skills - Rotation amongst MIUs and Acute A&E Minors to enhance practice and build relationships & confidence |
| Mental Health | - Hard to access service if between 16-18 - Can take time for patients to get access to the right service - Difficulties if patient is still deemed as not discharged clinically before MH team will get involved if patient in A&E. - MH beds - Waiting times for Psych appointments may cause deterioration in patient’s condition resulting in urgent attendance at A&E - Large caseload for MH Nurses in Community – fluctuating depending on location | - A&E Liaison located within A&E depts. - Signposting improved | - Single point of access for urgent mental health response - Access through 1 number and will be in operation in 3 centres covering Kent - Link to 111 - Routing and signposting - Tele-triage and assessment - Access to electronic patient record - Availability of SPA for relatives and carers as well as patients. | - Listening and empathy skills for SPA operators - More trained MH nurses in Community |
