# Supplementary material for: Using systems thinking to identify workforce enablers for a whole systems approach to urgent and emergency care delivery: a multiple case study
Source: BMC Health Serv Res. 2016 Aug 9;16:368. doi: 10.1186/s12913-016-1616-y (PMC4979146; doi:10.1186/s12913-016-1616-y)
Supplement: Additional file 6: Figure S1. — Levels of data analysis. (DOCX 50 kb) [file 12913_2016_1616_MOESM6_ESM.docx]

**Dataset 1**

Literature review to set urgent and emergency care context and identify stakeholders

**Primary level analysis**

Research team Inductive thematic analysis

**Dataset 2**

Urgent and emergency care stakeholder events using claims, concerns and issues and values clarification activity

**Secondary level analysis**

Triangulation of themes and deductive synthesis in relation to systems components

**Primary level analysis**

Collaborative inductive thematic analysis

**Primary level analysis**

Research team Inductive thematic analysis

**Dataset 3**

Online survey of the underrepresented stakeholders at the events

**Primary level analysis**

Research team Inductive thematic analysis

| **Key** | |
| --- | --- |
|  | Direction of influence of the outcomes of one method on a later method |
|  | Primary level data analysis |
|  | Secondary level data analysis |
