# Supplementary material for: Using systems thinking to identify workforce enablers for a whole systems approach to urgent and emergency care delivery: a multiple case study
Source: BMC Health Serv Res. 2016 Aug 9;16:368. doi: 10.1186/s12913-016-1616-y (PMC4979146; doi:10.1186/s12913-016-1616-y)
Supplement: Additional file 7: Figure S2. — Framework for achieving whole systems urgent and emergency care. (DOCX 34 kb) [file 12913_2016_1616_MOESM7_ESM.docx]

**Inputs Outputs**

**System Enablers**

- Whole pathway commissioning- integrated information & funding systems
- Interdependent partners across primary, secondary & tertiary care
- Leadership, expertise and collaborative ways of working
- Staff recruitment and retention strategies that attend to competence, role clarity, empowerment and support
- Public information for navigating the system

**Outcomes**

- Timely care at time of crisis in the right place
- Urgent and high dependency care prevents loss of life or on-going illness
- Consistent approach to care delivery experienced across regional communities and population
- Positive work based culture that enables person-centred, safe & effective care
- Improvements in mortality and quality outcomes
- Effective use of financial resources through reducing duplication of effort

**Integrated urgent and Emergency care**

**(Whole System)**

**Specific Workforce Enablers**

### Clinical systems Leadership

### Single career and competence framework (Assess Treat SORT)

### Work based facilitator of learning, development and improvement

### Curriculum content for High Education Institutions and Further Education Colleges

**Feedback**
