# Supplementary material for: Using systems thinking to identify workforce enablers for a whole systems approach to urgent and emergency care delivery: a multiple case study
Source: BMC Health Serv Res. 2016 Aug 9;16:368. doi: 10.1186/s12913-016-1616-y (PMC4979146; doi:10.1186/s12913-016-1616-y)
Supplement: Additional file 8: Table S1. — System enablers for a whole system approach to urgent and emergency care delivery. (DOC 32 kb) [file 12913_2016_1616_MOESM8_ESM.doc]

**Additional file 8: Table S1 System enablers for a whole system approach to urgent and emergency care delivery**

| **System enablers** | **Elements** | | |
| --- | --- | --- | --- |
| Commissioning | - Whole pathway commissioning- integrated funding and information systems to enable data sharing | | |
| Interdependent partners | ***Primary partners***  Providers of basic level of healthcare including health promotion, early diagnosis and prevention of disease. | ***Secondary partners***  Providers of intermediate level of healthcare performed in an environment with specialized equipment and laboratory facilities. | ***Tertiary partners***  Providers of highly specialized healthcare in specialist health centers usually on referral from primary or secondary health care providers |
| Leadership- clinical expertise and ways of working | - Systems leadership that models and drives integration across boundaries with expertise in culture change and enabling effective teams - Increases access to clinical expertise across the system - Develops a learning culture and facilitates an integrated approach to learning, development, improvement and evaluation - Facilitates collaborative inter-professional teamwork and partnership working - Aims for continuity of service across different contexts | | |
| Staff recruitment, competence, role clarity, empowerment and support | - Appropriate staff levels, role clarity, competence development and use - Strategy to grow and retain staff for a stable workforce through: - Career progression framework - Learning and development incentives - Appropriate empowering for decision making - Commitment to using the workplace as the main resource for learning, development and improvement - Strong administrative expertise and support | | |
| Public information and understanding | - Public education and information to navigate the system | | |
