# Supplementary material for: Using systems thinking to identify workforce enablers for a whole systems approach to urgent and emergency care delivery: a multiple case study
Source: BMC Health Serv Res. 2016 Aug 9;16:368. doi: 10.1186/s12913-016-1616-y (PMC4979146; doi:10.1186/s12913-016-1616-y)
Supplement: Supplementary file 9 [file 12913_2016_1616_MOESM9_ESM.docx]

Assess

- Assessment of first aid needs
- Holistic assessment skills at appropriate level
- Protocol driven assessment
- Using assessment tools
- Prioritisation decision making (Triage)
- Communication
- Risk assessment
- Information technology skills
- Self awareness

Treat

- Diagnosing in different settings
- Interpreting diagnostics
- Documenting
- Providing advice
- Prescribing (patient group directions)
- Delivering interventions in different settings
- Access to resources
- Decision making
- Interdisciplinary collaboration

SORT

- Understand the whole system
- Referral system/appropriate follow up
- Timely follow-up discharge
- Reflecting and evaluating
